# Perinatal autopsy in Ghana: Healthcare workers knowledge and attitude

**DOI:** 10.3389/fgwh.2022.1021474

**Published:** 2022-12-14

**Authors:** Alim Swarray-Deen, Dzifa A. Attah, Promise E. Sefogah, Nana E. Oduro, Hanson G. Nuamah, Mercy A. Nuamah, Catherine Adzadi, Samuel A. Oppong

**Affiliations:** ^1^ Department of Obstetrics & Gynaecology, University of Ghana Medical School, Accra, Ghana; ^2^ Department of Psychiatry, University of Ghana Medical School, Accra, Ghana; ^3^ Department of Obstetrics & Gynaecology, Korle Bu Teaching Hospital, Accra, Ghana; ^4^ Department of Epidemiology & Disease Control, School of Public Health, University of Ghana, Accra, Ghana

**Keywords:** autopsy, consent, decision, stillbirth, perinatal, post-mortem, mixed method

## Abstract

**Background:**

Perinatal mortality refers to stillbirths and early neonatal deaths. Stillbirth, the death of a foetus from 28 weeks or with a birth weight 1,000 g or above, and early neonatal deaths, the death of a new-born within 24 h of delivery, are among the most distressing global health problems, with approximately 2 million stillbirths occurring annually. Although a post-mortem examination of the stillborn baby is essential for understanding and learning the cause of stillbirth, many couples decline the procedure. Sub-Saharan Africa has one of the highest stillbirth rates in the world, yet there is a dearth of studies on post-mortem uptake from the region.

**Aim:**

To explore healthcare professionals' views and perceptions of perinatal autopsy in Ghana.

**Methods:**

Mixed-method approach consisted of semi-structured interviews and an electronic cross-sectional survey to evaluate the views and perceptions of healthcare professionals at Korle-Bu Teaching Hospital on autopsy for stillbirths and early neonatal deaths. Descriptive quantitative data were summarised in frequencies and percentages, and statistical results and descriptions were tabulated and coded in terms of types of barriers. For the qualitative aspect, the audio-taped interviews were transcribed, themes generated, and direct quotes and descriptions were coded for all knowledge, beliefs, attitudes and practices concerning the barriers and facilitators for post-mortem.

**Results:**

Ninety-nine healthcare professionals participated. No participant had formal training regarding counselling for perinatal autopsy and 40% had “***no idea***” who is responsible for counselling and obtaining consent for a perinatal autopsy. Forty-four percent (44%) of the participants knew of only the “Conventional/ Full” autopsy and <4% were aware of less invasive methods of performing an autopsy. Qualitative data showed healthcare worker influence, religious and financial considerations impede the implementation of perinatal autopsies. Despite the low uptake of perinatal autopsies, interviews from healthcare workers suggest acceptance rates would improve if parents knew about different options, especially less invasive procedures.

**Conclusion:**

At Ghana's largest referral centre, perinatal autopsy counselling and uptake are at extremely low levels. Most healthcare professionals have little knowledge, skills, and capacity to advise parents regarding perinatal autopsies. Training is needed to update the workforce on recommended perinatal autopsy practices.

## Introduction

Perinatal mortality, according to the World Health Organization (WHO), is the death of a child between 28 weeks of gestation and before the seventh day of life (1). This is a significant public health concern, with an estimated 2 million stillbirths and 1.8 million early neonatal deaths reported in current literature (2, 3). However, these numbers may be underestimated, as some are often under-reported (2).

Low- and middle-income countries share the highest burden, with over three-quarters of estimated stillbirths in 2019 occurring in sub-Saharan Africa and Southern Asia alone (42 per cent and 34 per cent, respectively) (2). A recent meta-analysis of data from sub-Saharan Africa reported an overall perinatal mortality rate of 34.7 per 1,000 births (4), which is 30% higher than the global estimate (5).

Identifying the cause of perinatal death is essential to provide recurrence risk assessment, establish implications for family members, and inform management of future pregnancies (6). Perinatal autopsy (including placental examinations) remains the gold-standard procedure to establish the presence or absence of foetal or neonatal abnormalities. This combined examination is the single most useful investigation in providing information to parents about why their foetus or new-born died (7, 8). Despite this, uptake remains well below the recommended 75%, even in developed nations (4, 9). Sub-Saharan Africa has one of the highest perinatal mortality rates in the world, yet there is a dearth of studies on perinatal post-mortem uptake from the region.

Ghana introduced a “Free” Maternal Health Care Policy in 2008 that enrolls pregnant women on the National Health Insurance Scheme to access antenatal, delivery and postnatal care free of charge. The policy's objective was to eliminate the financial barrier to access, thereby increasing antenatal attendance, facility delivery and postnatal care uptake for nursing mothers with their newborn babies for up to 90 days. Unfortunately, this package does not include the charges for perinatal autopsies.

Identifying the reasons why there are few requests submitted for perinatal post-mortem examinations from a professional perspective is vital to understand whether these might be mitigated by the introduction of less invasive methods and provision of institutional funding.

The objective of our study was to evaluate healthcare professionals' views and perceptions of perinatal autopsy in Ghana. This involved determining the participants' level of awareness of the post-mortem procedure and consent process, identifying barriers and motivators to perinatal post-mortem examination as well as assessing factors influencing uptake of a perinatal post-mortem examination.

## Materials and methods

This study used a mixed method approach using semi-structured interviews and an online cross-sectional survey to evaluate the views and perceptions of healthcare professionals on autopsy for stillbirths and early neonatal deaths at the Obstetrics Department of the Korle-Bu Teaching Hospital (KBTH), the largest tertiary referral hospital in Ghana. The department serves mainly as a referral centre for the southern part of the country, with an average of 10,000 deliveries per year, with nearly 80% of parturients being referred in labour. In 2020, the department recorded 31 per 1,000 perinatal death rate, with only a 1% perinatal autopsy rate.

At Korle Bu Teaching Hospital, placenta examinations for stillbirths are not done routinely, and autopsies are performed by the certified Pathologists in the Pathology Department. However, cases that required advanced analyses, such as immunohistochemistry, culture and sensitivity, and toxicology were outsourced from private laboratories at additional costs to the parents.

The list of healthcare professionals working (HCW) in the Obstetrics department was obtained from the Human Resource department, and each HCW was sent a link to the questionnaire *via* email or text message. Using simple random sampling, we selected 12 of those who consented to participate in the interviews.

The survey for the healthcare professional was designed to obtain information on the participant's socio-demographic characteristics, knowledge of the hospital's post-mortem procedure, and views and beliefs. One of the research team members, a clinical psychologist, conducted the qualitative interviews. This member of the research team, works as a private clinician outside of the Korle Bu Teaching Hospital, and has a background in qualitative research. The semi-structured interviews were held at the participants' convenience either in person or *via* Zoom, depending on preference. All interview sessions were conducted and recorded with the permission of the participants. The interview covered factors influencing post-mortem requests, the amount of information given to parents to help make post-mortem decisions, and the perceptions of the HCW about perinatal post-mortem. After data collection, each audio recording was transcribed verbatim and analyzed using thematic analysis. Thematic analysis is an effective method of comparing the perspectives of diverse research participants, highlighting similarities and differences, and generating unexpected insights (10). To obtain codes, the interview transcripts were read and reread. These codes were then categorized based on their similarity to produced themes. We compared these themes to the data and looked at them to see if they accurately reflected the text.

Quantitative data from the survey were exported into Stata 16.1 (StataCorp, College Station, Texas, USA) for analysis. The categorical variables are presented as frequency and percentages. The differences between demographic characteristics and participant's response were assessed with Fisher's exact test and *p*-values <0.05 were considered significant.

## Results

At the time the study was conducted, there were 324 active healthcare workers in the department and 99 (30.6%) responded to the online survey. The participants were from 5 different units within the department with characteristics shown in Table 1.

**Table 1:** Characteristics of study participants.

Characteristics	Frequency	%
Gender		
Female	57	57.6%
Male	40	40.4%
Not Stated	2	2.0%
Age		
25–29 years	29	29.3%
30–34 years	30	30.3%
35–39 years	23	23.2%
>40 years	13	13.1%
Not Stated	4	4.1%
Unit		
Children's ward	3	3.0%
Gynecology	8	8.1%
Maternity	51	51.5%
NICU	22	22.2%
Recovery	14	14.2%
Not Stated	1	1.0%
Job Classification		
Doctors (*n* = 64)^a^		
Consultants (Ob/Gyn)	4	4.0%
Specialists (Ob/Gyn)	7	7.1%
Residents (Ob/Gyn)	37	37.4%
House Officers	13	13.1%
Pediatricians	3	3.0%
Nurses (*n* = 35)^a^		
Critical care nurses	4	4.0%
Senior Staff Midwives	4	4.0%
Senior Staff Nurses	5	5.0%
Staff Midwives	7	7.1%
Staff Nurse	15	15.1%
Years of work		
1–5 years	58	58.6%
6–10 years	22	22.2%
10–15 years	13	13.1%
>15 years	4	4.1%
Not stated	2	2.0%
Relationship status		
Divorced	1	1.0%
Engaged	4	4.1%
Married	48	48.5%
Single	43	43.4%
Not Stated	3	3.0%
Religion		
Christian	92	92.9%
Muslim	5	5.1%
Not Stated	2	2.0%

^a^ Out of the 324 active healthcare workers in the department (149 nurses and 175 doctors) and 99 (30.6%) responded to the survey (35 nurses and 64 doctors).

More doctors (*n* = 64, 65%) than nurses (*n* = 35, 35%) participated in the survey. Fifty percent of the participants worked in the Maternity unit of the department, and there were more women (*n* = 57, 59%) than men (*n* = 40, 41%) who participated. The distribution was similar to the hospital employee demographics.

The number of years of professional experience was similar between the nurses/midwives and doctors, with more than 50% of participants from each group having been working in the profession for less than 6 years, and 17% in both groups having more than 10 years of professional experience (Supplementary Table).

The study found that gender and relationship status were statistically significant concerning the issue of a perinatal autopsy. Female participants were more likely to appreciate the sensitivity of the matter and aligned to counselling being done in a timely and accurate manner” than male participants (*p* ≤ 0.01). Married respondents were more likely to respond negatively to “perinatal autopsy, as an essential part of investigating stillbirths” as compared to single mothers (*p* ≤ 0.01). All other demographic characteristics did not significantly differ to the responses obtained (*p* > 0.05) (Table 2).

**Table 2:** Association between participants demographic characteristics and attitude towards perinatal autopsy.

	Demographic characteristics	Yes, *n* (%)	Neutral, *n* (%)	No, *n* (%)	*p*-value
Is perinatal autopsy an essential part of investigating stillbirths and neonatal deaths in your opinion?	Gender	Female	39 (68.42)	4 (7.02)	14 (24.56)	0.38
		Male	23 (57.50)	6 (15.00)	11 (27.50)	
	Years of work	1–5 years	36 (62.07)	7 (12.07)	15 (25.86)	0.84
		6–10 years	16 (72.73)	1 (4.55)	5 (22.73)	
		10–15 years	8 (61.54)	1 (7.69)	4 (30.77)	
		>15 years	2 (50.00)	1 (25.00)	1 (25.00)	
	Relationship status	Divorced	0 (0.00)	0 (0.00)	1 (100.00)	**0.01**
		Engaged	1 (25.00)	3 (75.00)	0 (0.00)	
		Married	31 (64.58)	2 (4.17)	15 (31.25)	
		Single	30 (69.77)	5 (11.63)	8 (18.60)	
	Religion	Christian	59 (64.13)	10 (10.87)	23 (25.00)	0.44
		Muslim	2 (40.00)	0 (0.00)	3 (60.00)	
		None	1 (100.00)	0 (0.00)	0 (0.00)	
Do you think it should be compulsory to do the counselling in the presence of both parents?	Gender	Female	37 (64.91)	11 (19.30)	9 (15.79)	0.17
		Male	19 (47.50)	9 (22.50)	12 (30.00)	
	Years of work	1–5 years	34 (58.62)	12 (20.69)	12 (20.69)	0.69
		6–10 years	13 (59.09)	3 (23.08)	6 (27.27)	
		10–15 years	6 (46.15)	3 (23.08)	4 (30.77)	
		>15 years	2 (50.00)	2 (50.00)	0 (0.00)	
	Relationship status	Divorced	0 (0.00)	0 (0.00)	1 (100.00)	0.55
		Engaged	3 (75.00)	1 (25.00)	0 (0.00)	
		Married	25 (52.08)	11 (22.92)	12 (25.00)	
		Single	27 (62.79)	8 (18.60)	8 (18.60)	
	Religion	Christian	53 (57.61)	20 (21.74)	19 (20.65)	0.26
		Muslim	2 (40.00)	0 (0.00)	3 (60.00)	
		None	1 (100.00)	0 (0.00)	0 (0.00)	
Do you think the counselling should be done in a timely and accurate manner in a quiet environment, allowing sufficient time to answer questions?	Gender	Female	23 (40.35)	4 (7.02)	6 (10.53)	**0.01**
		Male	16 (40.00)	6 (15.00)	3 (7.50)	
	Years of work	1–5 years	44 (75.86)	2 (3.45)	12 (20.69)	0.87
		6–10 years	17 (77.27)	0 (0.00)	5 (22.73)	
		10–15 years	9 (69.23)	0 (0.00)	4 (30.77)	
		>15 years	4 (100.00)	0 (0.00)	0 (0.00)	
	Relationship status	Divorced	0 (0.00)	0 (0.00)	1 (100.00)	0.19
		Engaged	4 (100.00)	0 (0.00)	0 (0.00)	
		Married	36 (75.00)	0 (0.00)	12 (25.00)	
		Single	34 (79.07)	2 (4.65)	7 (16.28)	
	Religion	Christian	72 (78.26)	2 (2.17)	18 (19.57)	0.22
		Muslim	2 (40.00)	0 (0.00)	3 (60.00)	
		None	1 (100.00)	0 (0.00)	0 (0.00)	

Bold values were found to be statistically significant.

### Knowledge of protocols & consent process

**Figure 1:**
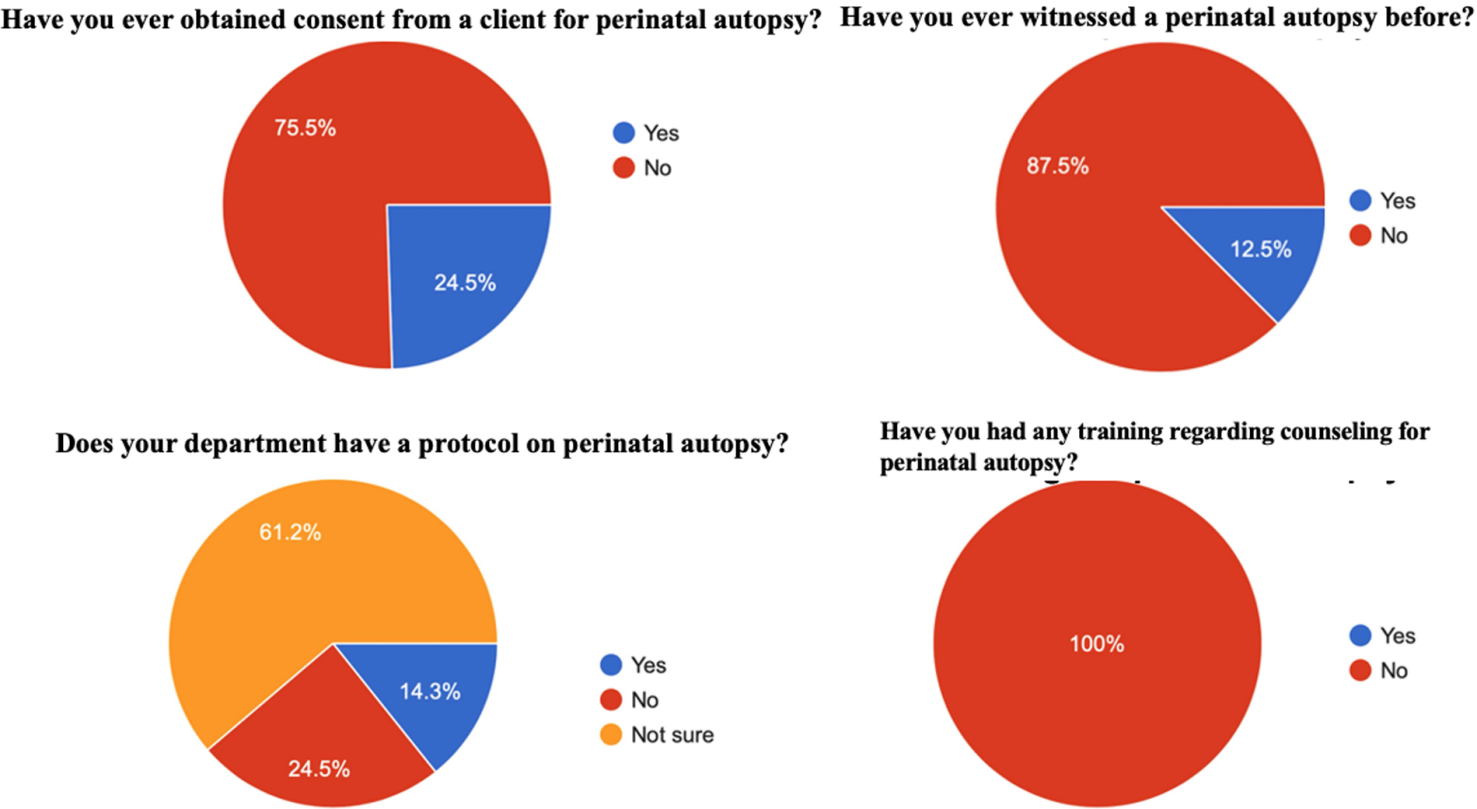
Summary of healthcare workers prior exposure to perinatal autopsy from survey.

No participant had received any formal training regarding counselling for perinatal autopsy in the department. Approximately 40% of respondents had “***no idea***” who is responsible for counselling and obtaining consent for a perinatal autopsy. Regarding the protocol for seeking consent from parents, 85.7% of the healthcare workers were “not aware” of an existing protocol in the department that offers guidance on how to handle the events concerning perinatal deaths (Figure 1). As the following quote demonstrates, some healthcare professionals are unfamiliar with the protocol and procedures for conducting a perinatal autopsy.

*“There is none, there is none, I don’t think so. Well, when I got in the department, I wasn’t taken through any process or any procedure in doing it” (HCW 13, Resident)*

Although a thorough orientation on the work process flows and clinical management are done for staff in the department for handling perinatal deaths, it does not seem to cover the protocol for conducting perinatal autopsies as illustrated in the quote below:

*“Honestly, the protocol for orienting new staff concerning perinatal autopsy, I am not too sure. But usually, before we start, an orientation is done for us which covers some of the basic issues that we may encounter in the department and then how to go about it.” (HCW 12, Resident)*

#### Previous participation in consent/autopsy process

Despite having limited knowledge of the protocol for conducting a perinatal autopsy, nearly a quarter of all respondents (24%) had witnessed or participated in the consent process for a perinatal autopsy. Among the participants, 12.5% had participated in a perinatal autopsy in the past 5 years. Figure 1 demonstrates the level of experience with perinatal autopsy within this study population. There appeared to be no formal training on how to obtain consent for an autopsy. Evidence from the qualitative data shows that some doctors usually observe what others do and replicate the process when the need arises. For instance, Healthcare worker 8 shared that:

*“I think, it is not done officially, they come in, they see it being done, then they also follow suit” (HCW 8, Specialist)*

#### Timing of counseling and need for the partner involvement

**Figure 2:**
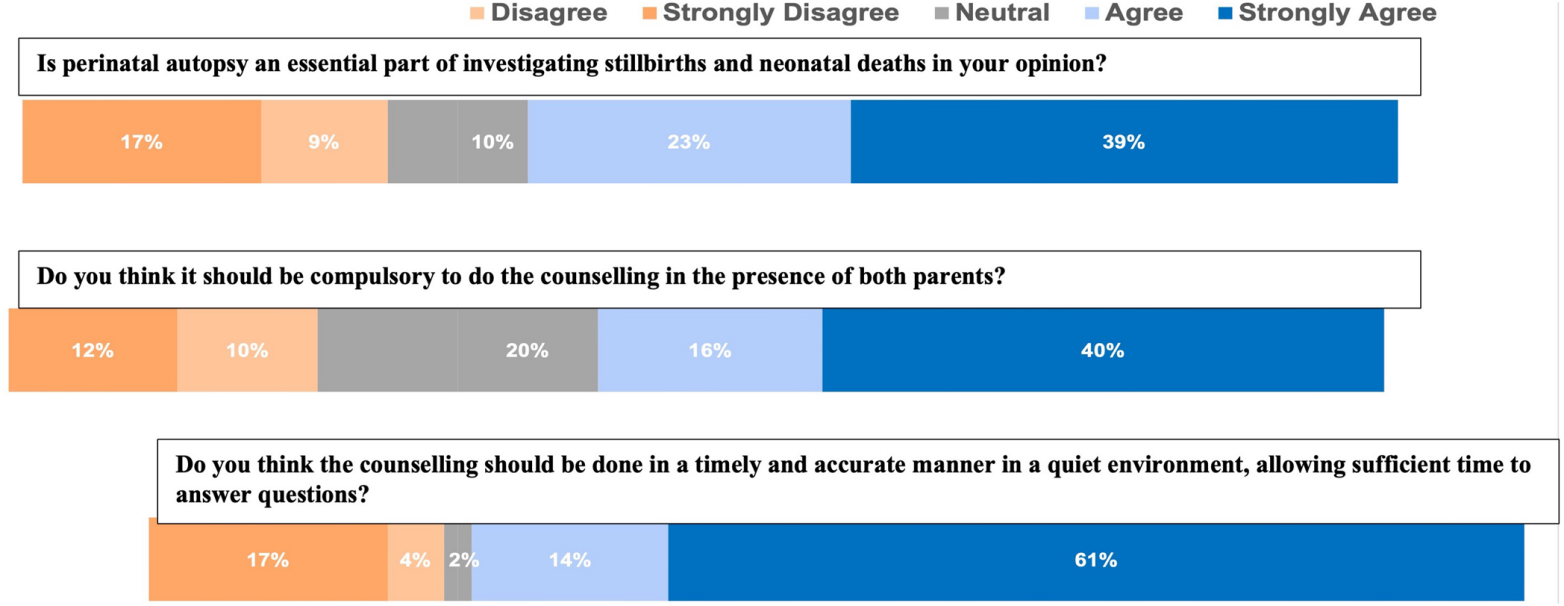
Summary of healthcare workers attitude towards perinatal autopsy from survey.

We also assessed healthcare workers' opinions on the relevance of having the partners of mothers who have delivered a stillbirth present at the time of counselling. Twenty-nine percent agreed that having the partners around was necessary and approximately 45% were not sure what the role the partners played in the consent process (Figure 2) During the qualitative interviews, some doctors believed the discussions on autopsy could be done immediately after the death declaration.

*“It depends, but sometimes it follows after the declaration, especially with those who perinatally, diagnoses were made and they’ve been counselled.” (HCW10, Resident)*

Conversely, some doctors believed it was best practice to give some time for parents to process the news of their baby's passing before bringing up the topic of an autopsy.

*“You need to let them get to terms with what has happened before you introduce the topic of having a post-mortem. It's not done immediately” (HCW 8, Specialist)*

*“If someone has lost a baby, I don’t think the day you break it to them should be the day you request the autopsy … if they are not going home that day, you can give them some one or two days, then you can discuss …” (HCW 5, House officer)*

#### Knowledge of the different types of autopsies

**Figure 3:**
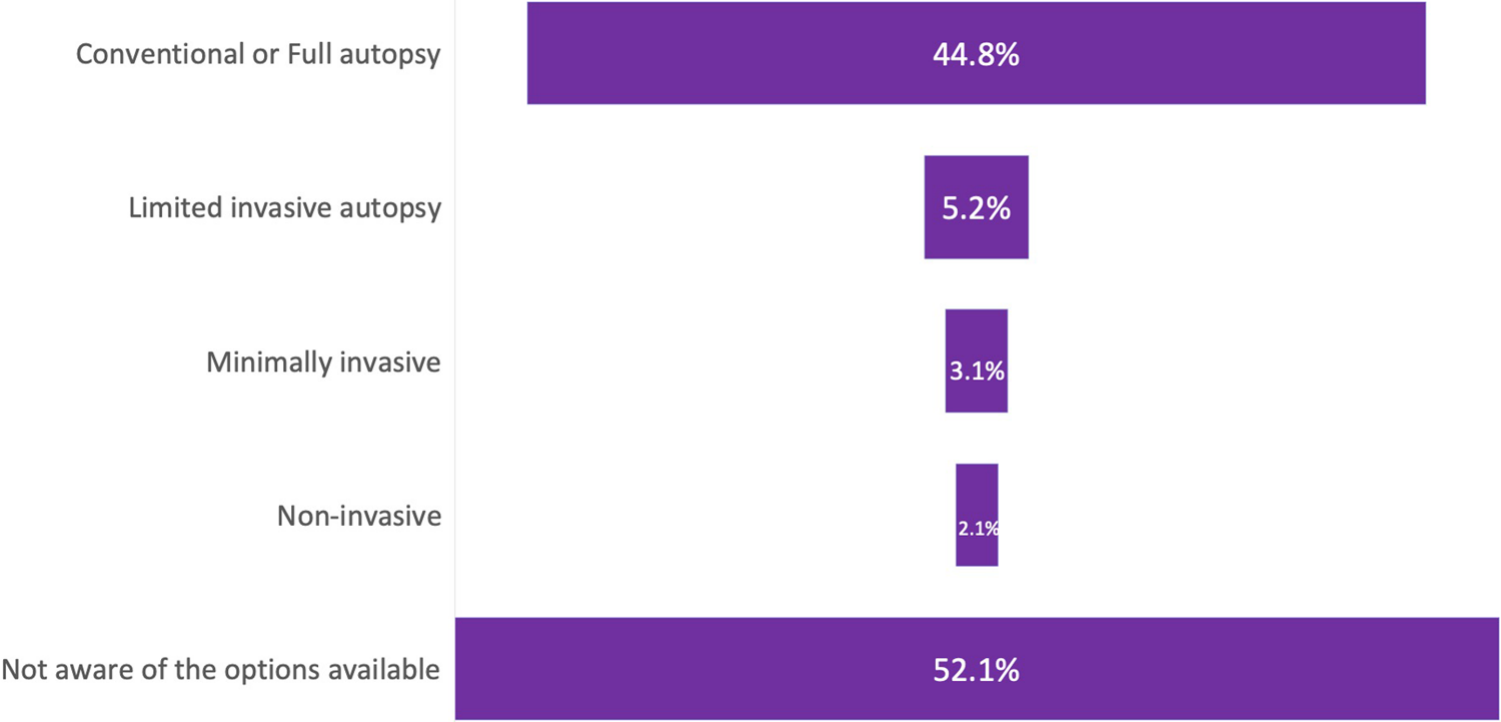
Responses to: “What type of autopsies have you requested for/or are aware of?”.

Knowledge of participants on different types of autopsies was limited, with 52.1% of respondents having no idea about the various perinatal autopsy examination modalities available, while 44% were aware of only the “Conventional/ Full” autopsy. Less than 4% were aware of the less invasive methods of performing an autopsy (Figure 3).

The interviews collected information on the different views the doctors had about types of autopsies as well as other information related to perinatal autopsies. With regards to the most suitable type of autopsy, there was no consensus. Some felt Minimally Invasive Tissue Sampling (MITS) was a better option because of its aesthetic appeal:

*“I think it is a good and the best procedure because if it is minimally invasive, you are not going to be doing a lot of cutting…so more like if it's going back to parents, they wouldn’t see much changes on the body” (HCS 10, Specialist)*

*“I think it is better than the full opening because after everything maybe the family would want to have … not like a funeral, but maybe a small gathering and the mother would want to look at the baby again before it is buried. Even though when they open, they close up, I don’t think they would be as meticulous as a surgeon” (HCW 5, House officer)*

Other doctors, however, felt that the choice of autopsy should be dependent on the answers it can provide. To these doctors, a full autopsy was preferred as it could give more answers:

*“The issue I have with that is it will not give you a full idea of what is going on. I think the full autopsy is better” (HCW 4, House officer)*

*“If we will be able to get enough tissue. Because even for adults … sometimes the report comes back saying we didn’t get enough tissue, that the tissue wasn’t enough for diagnosis” (HCW 11, Resident)*

#### Factors influencing autopsy uptake

The qualitative findings from this present study indicate that perinatal autopsies are never performed without parental consent. However, the participants narratives illustrates that doctors, as opposed to parents, make the request the majority of the time. HCS 3, a consultant stated that

“…..* rarely will parents request for autopsy…very rare. I can say that so far, I’ve not come across any situation that the parents want an autopsy” (HCW 3, Consultant)*.

A request for an autopsy does not necessarily result in the autopsy being performed, as a number of known barriers limit the utilization of perinatal autopsies. One such barrier is the parents' religious orientation. Some doctors believed that parents probably would not agree to an autopsy for their dead child because of their religious beliefs. The following participant quotes, illustrate the influence of religion:

*“… the religious beliefs of the patients … there are some that may believe that yes, the (2x) they’ll prefer their babies to go back to the maker intact.” (HCW 8, Specialist)*

*“… errr some Muslim family … I mean came with the … I mean we had the baby that was syndromic, we think that some investigations had to be done and they didn’t consent to it, they say they are going to bury the body in the same day so it was also difficult” (HCW 13, Resident)*

The cost of conducting an autopsy is another factor affecting autopsy requests, as shown in the following quote:

*“So, I think one of them would be cost … cost. I think most of the time, the autopsy we have to do is borne by the relatives, so I think cost may be one of the issues” (HCW 12, Resident)*

It appears from the qualitative data that autopsy costs are not always borne by the parents; sometimes the healthcare workers offset the expense to encourage patronage.

*“A lot of times, the cost goes to the bereaved. But there have been instances where if the cost is going to be a barrier, we will have to fund the post-mortem from our own coffers. The cost is for the parents. But if that is going to be a barrier, then we will go ahead and then fund the cost.” (HCW 8, Specialist)*

*“Most of the time, you know autopsy comes with a cost…errm, err so, but if we initiate it, the, it won’t come with a cost. So errm yeah. So that, so we assure them that it's not going to, come at any cost to them. I mean, also, errm, they can decline it” (HCW 3, Consultant)*

The low rate of autopsies is not solely the responsibility of parents. Occasionally, the healthcare workforce contributes to low uptake because of inaction of the healthcare worker or the cause of death is deemed known, as reflected in the quotes below:

*“For some cases, if you clearly know what caused the death then there will be no need for you to conduct an autopsy.” (HCW 5, house officer)*

*“I think sometimes too health staffs, we ourselves, like well, if we, I mean we usually have the death, the delivery, and that is it, we end it there. I think if we sat down and we started interrogating ourselves, what might be the cause of the death.” (HCW 13, resident)*

#### Training needs

As depicted in the section above, doctors are typically the ones who request consent, so the ability of doctors to persuade parents who have suffered perinatal losses to agree to an autopsy is crucial. As reflected in the quotes below, some participants believed that training on how to communicate the benefits of an autopsy to these grieving parents was necessary.

*“it [training]helps us to be educated on the importance of doing an autopsy and maybe the skill in convincing or taking consent from parents to do autopsy” (HCW 3, Consultant)*

In addition to knowing how to obtain parental consent for the autopsy, one doctor emphasized the need for training on how to communicate the results of the autopsy, see the quote below:

*“And also on the post autopsy counselling, after you’ve gotten the request, how to convey the information to the relatives and how to explain it to them to understand the issue that the autopsy revealed” (HCW 12, Resident)*

Some participants believed that training health professionals on the significance of conducting perinatal autopsies would be beneficial. Reflecting on the attitude of healthcare workers and training needs, one participant emphasized that:

*“They should be educated on the importance of perinatal autopsy. I am sure if you ask, some health workers are not really in support of it” (HCW 4, House officer)*

In addition to training in soft skills, some respondents believed it necessary to train more personnel to conduct autopsies, as this would accelerate the rate at which pathology unit reports are generated. One healthcare worker shared that:

*“I'm sure we also don't have a lot of pathologists that are specialised in perinatal autopsy, most of them will deal with it with adults and children but not necessarily neonates” (HCW 9, Specialist)*

Table 3 provides a summary of the major themes and sub-themes from the qualitative data.

**Table 3:** Themes, sub-themes and examples of healthcare workers’ quotes.

Themes	Sub-Themes	Quotes
**Autopsy Procedures**	**Poor knowledge of perinatal autopsy protocol**	*I am not really sure what goes into that. And I don’t even know the cost, that's another thing. So right now even if you give me…I don’t know what to tell about cost. I will have to find out. Then the duration…… (HCW 10, Resident*
	**Informal training**	*“I think, it is not done officially, they come in, they see it being done, then they also follow suit” (HCW 8, Specialist)*
	**Timing for making autopsy request**	*“If someone has lost a baby, I don’t think the day you break it to them should be the day you request the autopsy…if they are not going home that day, you can give them someone or two days, then you can discuss… ““ (HCW 5, House officer)*
	**Autopsy preference**	*“I think it is a good and the best procedure because if it is minimally invasive, you are not going to be doing a lot of cutting…so more like if it's going back to parents, they wouldn’t see much changes on the body” (HCW 10, Resident)*
**Factors influencing barriers to Autopsy Intake**	**Cost of autopsy**	*Some the moment you say oh, this is how, I mean they want to do autopsy, the first thing they ask is how much? (HCW 13, Resident)*
	**Religious beliefs**	*…the religious beliefs of the patients…there are some that may believe that yes, the (2x) they’ll prefer their babies to go back to the maker intact. (HCW8, Specialist)*
	**Healthcare workers influence**	*I am sure the only place perinatal autopsy would be needed would be at NICU (HCW 4, House officer)*
**Training Needs**	**Autopsy procedures**	*“it [training] helps us to be educated on the importance of doing an autopsy and maybe the skill in convincing or taking consent from parents to do autopsy” (HCW 3, Consultant)*
	**Appropriately disclosing the autopsy results**	*“And also on the post autopsy counselling, after you’ve gotten the request, how to convey the information to the relatives and how to explain it to them to understand the issue that the autopsy revealed” (HCW 12, Resident)*

## Discussion

The benefits to gain from a thorough review of perinatal death span from improvements in clinical care to public health interventions for prevention. According to the WHO, determining the cause of a perinatal death helps as an indispensable aid to parents and clinicians wanting to understand why their baby died and to determine the recurrence risk and management in the subsequent pregnancy (11). Consequently, a proper diagnostic inquiry is needed after a perinatal death, including collecting pertinent information about the mother's medical history, obstetric history, placental examination, molecular cytogenetic analysis, and foetal autopsy (8).

In cases where parents do not consent to autopsy, alternative approaches such as minimally invasive post-mortem examination, post-mortem magnetic resonance imaging, and fetal photographs are good alternatives. After all investigations have been performed it is important to combine findings from the clinical review and investigations together, to identify the most probable cause of death and counsel the parents regarding their loss.

The ideal recommendation is a mortality audit in which information on the number and causes of stillbirth and early neonatal deaths is collected and subsequently used for systematic and critical analysis of the quality of care received. It is an established mechanism for investigating each death's circumstances, including treatment failures that could have been avoided. Utilization of the audit cycle to the circumstances encompassing perinatal deaths is an established quality improvement strategy that can emphasize breakdowns in clinical care at the local level as well as failures in processes at the district or national level. This process is already being used in many countries in the form of maternal death surveillance and response (MDSR) (12).

To the best of our knowledge, this is the first study from Ghana documenting the perceptions and attitudes of health professionals on the uptake of perinatal autopsy. The decision to go ahead with a perinatal autopsy depends on both the healthcare staff and the deceased infant's family. As such, steps to increase the rate of perinatal autopsy uptake should factor in the concerns and limitations of both parties. The present study focuses on the knowledge and attitude of healthcare professionals from a premier teaching hospital where various cadres of healthcare workers receive training, such as doctors, nurses and midwives at the undergraduate and postgraduate levels. This Teaching Hospital is also responsible for conducting and sustaining the perinatal autopsy within the existing system. At the time the study was conducted, there was a departmental protocol describing the steps for handling and transferring perinatal deaths, but limited information on the counselling process and options available. The Pathology unit can perform both a complete and limited autopsy, but advanced imaging, cytogenetic or molecular techniques were unavailable at the Korle Bu Teaching Hospital.

We found that the healthcare workers had no formal training on the counselling process for perinatal autopsy. According to them, the barriers to the uptake of perinatal autopsy were mainly due to religious and financial implications. The qualitative data generated by the healthcare worker interviews reinforced the quantitative findings.

The lack of specialised training among healthcare professionals to consent patients is a crucial issue in this study. Counselling and the consent process involve complex interactions between parents, family members, and a multitude of professionals, these processes are passively acquired skills.

This is a consistent theme even in High and Middle-Income Countries (HMICs) reporting low levels of training among healthcare workers (21%–37%) (13). A study by Epstein et al. showed that HCWs agreed that seeking consent was a difficult conversation to have with parents and expressed a strong desire amongst residents and specialists for more guidance and training in the process (14). The expressed need for training included an awareness of the need to pay attention to staff's emotional and psychological needs. Doctors in the present study mentioned that one area in which they needed to receive training was how to effectively disclose and discuss the autopsy with parents while being empathetic. As such, skills training on how to approach and discuss autopsies with parents, considering their emotional state, would help improve parent experiences and subsequently increase the rate of perinatal autopsy uptake (15).

As part of training on consenting, Lewis et al. demonstrated that professionals that had observed an autopsy had greater confidence in consenting parents and were more satisfied with their training (16). From our study, only 12 percent of respondents (all resident doctors) had ever witnessed a perinatal autopsy as part of their training, and such rotations are not even applicable to all concerned units at our facility.

One way to improve uptake is by understanding families' concerns about agreeing to autopsies (17). For instance, if the family's religious orientation causes them to turn down a complete autopsy because of the time involved and the incisions made to the body, having a conversation about non-invasive explorations could make them more amenable to the uptake of perinatal autopsy. Numerous studies have demonstrated that Jewish and Muslim parents would be more inclined to agree to a non-invasive procedure and could also delay burial by one or two days if they were well informed about the non-invasiveness of the proposed procedure and the benefits of the findings to future pregnancies (18, 19).

Regardless of religion, parents are more likely to agree to an autopsy that involves little or no incisions (6, 7). As such, in talking to parents or obtaining consent for an autopsy, it is necessary to bear this in mind and inform them about the different types of autopsies available. The uptake of PM has increased significantly amongst these religious groups in HMICs since the introduction of MITS and non-invasive autopsy (20, 21).

Our study demonstrated that the healthcare workers in direct contact with the bereaved parents had little knowledge of the various types of autopsies that could be offered. This may be due to the pathology unit's limited options or perinatal pathologists' limited availability to provide these specific services. Nonetheless, non-invasive or minimally invasive autopsies are being done in some LMICs with impressive uptakes since the formation of the MITS Surveillance Alliance in 2018 with funding from the Bill & Melinda Gates Foundation to expand pathology-based mortality surveillance and support the generation of improved CoD data (22). Successful MITS has been reported in Bangladesh, India, Kenya, Mali, Mozambique, Pakistan, and South Africa (23–27).

Doctors interviewed indicated they did not have enough information to obtain consent for an autopsy. For example, information such as who should perform the counselling, the cost involved, the time needed for the report to be ready, or what happens during the autopsy was lacking. This has been shown to limit autopsy uptake since ill-informed HCWs would not have the information needed in order to convince parents to consent to an autopsy. Therefore, it is necessary not only to equip doctors with the soft skills required to successfully hold a conversation on autopsy with parents but also to give them the opportunity for practical experience with autopsies. In addition, policies regarding the training and orientation of new staff on the procedures for seeking consent must be adhered to. It will also be beneficial if this orientation is not done only when the new personnel arrive in the unit but repeated periodically to keep staff updated on best practices (28).

Another concern raised from the study was the low number of pathologists and how it affected the speed of conducting the autopsy and preparing reports. To overcome this, it is necessary to train more staff, particularly in neonatal pathology, as the pathologists who conduct adult autopsies might sometimes be less conversant with the smaller equipment they have to use and therefore fail to do so (29).

The cost of autopsies was identified as a barrier to the uptake of post-mortem examinations from our study. In Ghana, while the free maternal healthcare policy covers antenatal, delivery and postnatal care costs, the costs of storage/preservation and autopsy in cases of perinatal deaths, as at the time of this study, were all borne by the affected parents. The results of our study also raised an ethical issue; should the doctors counsel and pay for the procedure based on their academic interests in the case, or these should be borne by the facility or the healthcare service.

### Limitation

We acknowledge the sample size may be limited for this important study but coming from the largest referral and teaching hospital in Ghana, this may be a reflection of the practice in the Ghanaian context. Notwithstanding, it revealed the key findings with implications for training, policy interventions, and financing issues. This will also set the foundation for other larger future studies on the subject of perinatal and non-invasive autopsies in Ghana.

Other limitations include; the study being carried out at a single institution which could make the results specific to that institution. Health Professionals at various institutions may have distinct perspectives and experiences. Secondly, few Consultant / Specialist Obstetricians participated in this study due to time constraints or rotation to a different clinical service. Thus, the senior-level perspective is under-represented in this study. Finally, there was no representation from the pathology unit in this study. Though they are responsible for performing the procedure, they are hardly called upon to seek consent. Nevertheless, they may be able to provide further insight into the barriers to the uptake of perinatal autopsies in our setting. Finally, understanding parents' perspective is important for autopsy counselling and uptake, but this was outside the scope of the present study as we focused on caregivers' perspective to understand the reason for the low numbers of perinatal autopsy requests. Parents' perspective is our objective for further studies and we agree with the recommendation for global implementation of the eight principles for stillbirth bereavement care and involvement of the voices of women and families (30).

### Strengths of the study

1. First report on perinatal autopsy in Ghana and hence provides useful preliminary data for engagement on the subject
2. Mixed method study offers a deeper understanding of the subject and relates observed practice with perspectives and rationale

## Conclusion

There is low level of perinatal autopsy counselling and uptake at Ghana's largest referral center. The majority of healthcare workers are limited in their knowledge, skills, and ability to counsel parents on perinatal autopsy. The knowledge gleaned from conducting perinatal autopsies helps prevent future neonatal deaths. Nonetheless, certain key factors contributed to the low uptake of autopsies in the hospital setting. Professional training on understanding parents' concerns, ability to obtain parental consent, explanation of different types of autopsies available, and having the requisite human resource to improve the turn-around time of autopsy conduct and reporting are key factors that can improve the rate of perinatal autopsy uptake.

The decision to go ahead with a perinatal autopsy depends on the deceased infant's family and healthcare staff. Therefore, there is a need to assess the associations between provider–parent relationships and continuity of care. Decisional and ethical conflict and communication patterns are potentially valuable for designing intervention strategies.

A multisite study of HCWs as well as the parental view on perinatal autopsy are the focus of future studies and would also broaden thematic findings.

## Recommendations

Based on findings from our study, the following are recommended:

• That the cost of perinatal autopsies in Ghana should be included under the free maternal care policy package's coverage to remove the financial barrier identified.
• Institutionalization of training for clinicians, nurses and midwives at the Obstetric and Newborn Units on counselling and referrals for perinatal autopsies at the Korle Bu Teaching Hospital.
• A more extensive multi-centred study be conducted to scientifically obtain and explore additional broad-based perspectives on the subject of a perinatal autopsy, including the views of Pathologists for a comprehensive documentation of all relevant perspectives to inform policy in Ghana and beyond.

## Data Availability

The original contributions presented in the study are included in the article/**Supplementary Material**, further inquiries can be directed to the corresponding author/s.
