# Corrigendum: Perinatal autopsy in Ghana: healthcare workers knowledge and attitude

**DOI:** 10.3389/fgwh.2023.1196549

**Published:** 2023-05-31

**Authors:** Alim Swarray-Deen, Dzifa A. Attah, Promise E. Sefogah, Nana E. Oduro, Hanson G. Nuamah, Mercy A. Nuamah, Catherine Adzadi, Samuel A. Oppong

**Affiliations:** ^1^ Department of Obstetrics & Gynaecology, University of Ghana Medical School, Accra, Ghana; ^2^ Department of Psychiatry, University of Ghana Medical School, Accra, Ghana; ^3^ Department of Obstetrics & Gynaecology, Korle Bu Teaching Hospital, Accra, Ghana; ^4^ Department of Epidemiology & Disease Control, School of Public Health, University of Ghana, Accra, Ghana

**Keywords:** autopsy, consent, decision, stillbirth, perinatal, post-mortem, mixed method

A Corrigendum on Perinatal autopsy in Ghana: healthcare workers knowledge and attitude By Swarray-Deen A, Attah DA, Sefogah PE, Oduro NE, Nuamah HG, Nuamah MA, Adzadi C and Oppong SA. (2022) Front. Glob. Womens Health 3:1021474. doi: 10.3389/fgwh.2022.1021474

**Text Correction**

In the published article, there was an error in the **Abstract**, *Background*, Paragraph 1. It previously stated: “Perinatal mortality refers to stillbirths and early neonatal deaths. Stillbirth, the death of a foetus from 28 weeks or with a birth weight below 1,000 g, and early neonatal deaths, the death of a new-born within 24 h of delivery, are among the most distressing global health problems, with approximately 2 million stillbirths occurring annually.”

The corrected sentence appears below:

“Perinatal mortality refers to stillbirths and early neonatal deaths. Stillbirth, the death of a foetus from 28 weeks or with a birth weight **1,000 g or above,** and early neonatal deaths, the death of a new-born within 24 h of delivery, are among the most distressing global health problems, with approximately 2 million stillbirths occurring annually.”

The authors apologize for this error and state that this does not change the scientific conclusions of the article in any way. The original article has been updated.

