# Supplementary material for: Perinatal autopsy in Ghana: Healthcare workers knowledge and attitude
Source: Front Glob Womens Health. 2022 Dec 14;3:1021474. doi: 10.3389/fgwh.2022.1021474 (PMC9794746; doi:10.3389/fgwh.2022.1021474)
Supplement: Supplementary file 1 [file Table1.docx]

Supplementary Table: Profession by years of experience

|  | Years of experience | | | | | | | | | |  |
| --- | --- | --- | --- | --- | --- | --- | --- | --- | --- | --- | --- |
|  | 1-5 years | | 6-10 years | | 10-15 years | | > 15 years | | Not Stated | | P - Value |
|  | n | (%) | n | (%) | n | (%) | n | (%) | n | (%) |  |
| Doctor | 35 | (56.5) | 16 | (25.8) | 8 | (12.9) | 3 | (4.8) | 1 | (1.6) | 0.87 |
| Nurse | 23 | (65.7) | 6 | (17.1) | 5 | (14.3) | 1 | (2.9) | 0 |  |  |
